# Teamwork and Patient Safety in Intensive Care Units: Challenges and Opportunities

**DOI:** 10.3233/SHTI220120

**Published:** 2022-06-06

**Authors:** You Chen, Yang Gong

**Affiliations:** a Vanderbilt University Medical Center, Nashville, TN, USA; b The University of Texas Health Science Center at Houston, TX, USA

**Keywords:** Intensive care units, Patient safety, Teamwork, Telehealth

## Abstract

Intensive Care Units (ICUs) are recognized as a susceptible area to potential errors resulting in adverse outcomes. Healthcare professionals are multi-tasking, information-overloaded, and often with an interruptive workflow in ICUs. Such a stressful work environment poses challenges to reach a shared mental model in clinical practice, which leads to ineffective communication and reduces their awareness of potential safety risks. Despite data sources or materials supporting patient safety and team training being available, little research has been conducted to measure teamwork in clinical practice and to detect inefficient communication factors. The advent of telehealth provides an opportunity for remote video watchers to observe the entire process of patient care and related team activities. The potential application of video analyzing algorithms to video recordings can detect safety risks retrospectively. This article presents major teamwork and patient safety challenges in ICUs, and the opportunities of utilizing available data and telehealth including video recordings in future patient safety and teamwork research.

## Introduction

Critically ill patients admitted to ICUs are heterogeneous in their underlying illnesses, comorbidities, and requirements for invasive or nearly continuous monitoring of physiologic and laboratory values [1]. Healthcare professionals manage various medical devices (e.g., physiological monitors, ventilators, infusion pumps, and bedside computer terminals) and a high volume of electronic health record data when they deliver care to their patients [1–2]. Clinicians in ICUs collaborate, communicate, and coordinate to exchange continuous patient information updates, form a shared mental model, and reach a consensus on the patient’s health status [2–3]. Also, it is not uncommon for ICU teams to simultaneously conduct multiple tasks or frequently switch from one task to another, which brings fractured attention [4]. The exchanging of continuous patient information and the increasing fractured attention leads to ineffective and inaccurate communication and error opportunities [2–4].

The demand for intensive care, when exceeding available resources (e.g., bed and staff capacity), exacerbates clinician workload and undivided attention, leading to cognitive or system errors [4–5]. Because of increasing information overload and clinicians’ fractured attention in ICUs, a shared mental model is fragile in clinical practice, leading to miscommunications and medical errors. The critical problem is teamwork executed in clinical practice is seldom systematically investigated [3], and thus little evidence on team factors (collaboration, communication, and coordination) leading to medical errors is available to optimize the ICU teamwork. The availability of patient safety research data resources and team monitoring strategies can be leveraged to measure teamwork and detect and prioritize weaknesses for patient safety enhancement.

ICUs equipped with telehealth systems are expected to reduce clinician workload and fractured attention, improve team communication efficiency, and enhance patient safety [6]. Relying on telehealth technologies, video watchers can observe and analyze live video streaming to identify potential safety risks during patient care that are hard to be realized by bedside clinicians and communicate with bedside teams timely to prevent safety issues. Moreover, video recordings and analysis results are excellent training and educational materials to improve team leadership, team communication, peer support, and situation awareness. The video data is promising in becoming mainstream materials for teamwork and patient safety research in the future.

There has been an urgent need to increase the adoption of advanced team models (e.g., telehealth) in ICUs to reduce bedside clinician information overload, promote effective and accurate data communication, and improve patient safety. This perspective discusses major patient safety challenges in ICUs, team training strategies, data sources to enhance patient safety, and the opportunities of telehealth including video recordings in future patient safety research.

### Main Patient Safety Challenges in ICUs

The most frequent patient safety events (PSEs) in ICUs are associated with diagnosis and medication use [7–8]. Diagnostic and medication errors represent a large portion of preventable morbidity and mortality in hospitalized critically ill patients.

*A diagnostic error* is defined as a missed, wrong, or delayed diagnosis by some subsequent definitive test or finding [7]. Diagnostic errors resulted in 40,000 to 80,000 deaths annually in the United States, among which one out of five deaths in ICUs was due to misdiagnosis [7]. Examples of diagnostic errors include undiagnosed infections (sepsis, spinal abscess, pneumonia, endocarditis), vascular events (stroke, myocardial infarction, venous thromboembolism), and congenital/metabolic conditions (neonates). According to the Nation Academy of Medicine, diagnostic error is regarded as a blind spot in patient safety [9]. Diagnostic errors may be largely unknown because diagnostic safety data are not easy to identify and explore. A systematic approach is essential to collect vast amounts of clinical and administrative data. However, it is still a big challenge to identify error signals for possible diagnostic errors from clinical and administrative data, which does not capture every patient care scenario. Cultivating a safety culture and promoting teamwork among interdisciplinary healthcare workers are highly suggested to improve timely and accurate diagnoses [2–3].

*A medication error* is an error at any step along the pathway that begins when a clinician prescribes a medication and ends when the patient receives it [8]. The inconsistency between medication prescribing and administration is most commonly identified across each population and mostly related to dosing errors. There are several types of medication errors, including *adverse drug events (ADE), omission*, and *commission*. ADE includes medication errors, adverse drug reactions, allergic reactions, and overdoses. It is harmful and occurs with alarming frequency in critically ill patients. The clinical outcomes of ADEs can result in end-organ damage and even death. Complex pharmacotherapy with multiple medications increases the probability of ADEs in critically ill patients. ADE reasons include but are not limited to drug dosing (because of complex diseases), vulnerability to rapid changes in pharmacotherapy, ICU environment, complex drug regimens, and the mode of drug administration, especially for high-alert medications. *Omission* error is characterized by a failure of action such as missed diagnosis, delayed evaluation, or failure to prescribe a required drug treatment. For example, hypoglycemia was documented and not treated. *Commission* error is characterized by an incorrect action, such as administering the wrong drug to the wrong patient at the wrong time. For example, phototherapy was prescribed for a newborn infant but was issued for another neonate. The occurrence of a medication error is common during the stage of prescribing transcription, dispensing, and monitoring. Overdosing for neonates is particularly common in ICUs. Duplication of therapy and unnecessary prescribing of medications are also common for adults or elderly patients.

To help clinicians improve their access to fragmented information and synthesize the medication and patient data during the medication workflow, *health information technology (HIT) systems*, such as computerized provider order entry (CPOE), medication reconciliation, automated transcription, dispensing cabinets, Bar-coding medication administration, have been designed and implemented to intercept preventable medication errors. Unfortunately, HIT systems, are regarded as a double-edged sword, introducing not only the perceived benefits but unexpected consequences such as increased clinician workload, concerns of clinicians’ well-being (e.g., stress, fatigue, and interruptions), and new types of PSE induced by HIT.

In addition to HIT systems, ICU *interdisciplinary teams* with a shared mental model in prescribing, transcription, dispensing, administration, and monitoring will help reduce avoidable medication errors [8]. A growing body of evidence of medication errors has been identified based upon data from EHR notes, CPOE alert overriding reasons, and PSE reports [10]. However, it is rare to observe evidence-based training toward a shared mental model between ICU team members.

### Teamwork: interprofessional collaboration, coordination, and communication

An Interprofessional health care system is recommended to communicate systematically and continuously to meet patients’ individual needs. High-performing teams communicate well, provide feedback to each other, understand each team member’s roles, anticipate each member’s needs and behaviors, manage conflict amongst each other, and support each other. Several team Tools and strategies are available to enhance team performance and patient safety. TeamSTEPPS was created to train team members to reach a shared mental model to reduce cognitive and system errors [11]. The development of TeamSTEPPS relied on 25 years of research related to teamwork, team training, and team culture and borrowed team management ideas from Crew Resource Management protocols in aviation [11]. TeamSTEPPS identified four core teamwork competencies: communication, leadership, situation monitoring, and mutual support to promote effective and accurate communication [11]. The application of TeamSTEPPS in ICUs has been reported successful for improving team communication, leadership, mutual support, and incident awareness, and subsequently reducing medical errors and improving patient safety.

Recently, the Department of Health and Human Services (HHS) and the Agency for Healthcare Research and Quality (AHRQ) released strategies for reducing medical errors and enhancing patient safety [12]. The strategies highlighted the achievements made by the Comprehensive Unit-based Safety Program (CUSP), a method that helps clinical teams make care safer by combining improved teamwork, clinical best practices, and the science of safety. CUSP focuses on improving safety culture, teamwork, and communication, together with a set of evidence-based technical interventions, such as a checklist. The Core CUSP toolkit is modular and adaptable to meet individual unit needs. Each module includes teaching tools and resources to support change at the unit level. The CUSP toolkit has been applied to prevent central line-associated bloodstream infections (CLABSIs) in ICU patients. Among the 1,800 adult ICUs using the CUSP toolkit, CLABSIs were reduced from a baseline of 1.9 infections per 1,000 line days to a rate of 1.1 infections, or a 41% reduction [12].

Although CUSP toolkit and TeamSTEPPS aim to train and educate clinicians’ team skills, it remains challenging to measure the differences between the executed and trained/planned teamwork. As a result, limited evidence is available for healthcare organizations or care teams to prioritize the improvement in teamwork. For instance, clinicians are trained to have a shared mental model while the shared mental model may be broken in clinical practice. Although novel models have been developed to measure ICU teamwork [13–15], teamwork in practice is not well monitored and measured due to the lack of available infrastructures and patient safety data. Thus, little evidence can be leveraged to detect inefficient communication between teammates and reasons breaking the shared mental model.

### A patient safety event involving ICU teamwork

A medication error that occurred in an adult ICU may help us illustrate the importance of monitoring the differences between the actual/executed teamwork and trained/planned teamwork.

An adult patient admitted to the ICU was found confused with severe hyponatremia (sodium 109mEq/L, normal range 135mEq/l). At 7:30 am, a nephrology consultant reviewed the laboratory results and asked an intensivist to administer hypertonic saline (3% sodium) immediately to increase the sodium level, and to recheck the sodium level in one hour. He did not specify how much hypertonic saline should be administered. When the nephrologist came to the ICU at 9:30 am, the patient’s confusion had not improved. His sodium had risen to 130 mEq/dL, a rapid increase that put the patient at risk of severe neurologic complications. The nephrologist noticed that a 500 mL bag of hypertonic saline had nearly finished infusing. The infusion was stopped immediately, and the patient was administered medications to mitigate the effect of the rapid sodium correction. Fortunately, the patient’s sodium stabilized and his mental status gradually improved. [16]

Further investigation on this PSE revealed that the intensivist had intended to order administration of 50 mL of 3% saline. However, the default intravenous fluid order in the hospital’s computerized order entry system was for a 500 mL infusion. A separate, customizable order was available but not easily accessible. In a rush, the intensivist ordered the 500 mL infusion and added a free-text comment to “infuse 50 mL then recheck sodium.” Unfortunately, the free-text comment was missed by the pharmacist and ICU nurse, resulting in the patient receiving a much larger infusion at a faster rate than intended. The PSE revealed miscommunication mediated by Health IT between the nephrology consultant and the intensivist (50 mL to 500 mL), and those between the intensivist and the nurse (without specifying the time the sodium level should be rechecked). The intensivist and the nurse had not realized such an incident until the nephrology consultant’s revisit.

Therefore, effective communication among all patient team members is critical for understanding the timely start and discontinuation of therapy to achieve optimal clinical targets. Although Interprofessional tools such as TeamSTEPPS aiming to train and educate clinicians’ team skills, improving collaboration, communication, and coordination in ICUs, the details on how teamwork is executed in clinical practice are largely unknown due to the challenges of measuring team communications in clinical practice. As a result, the situation posits the challenge that miscommunication is not realized by ICU managers and healthcare organizations. The data sources such as EHRs, patient safety reports, do not usually capture the complete picture or details of communications between teammates and thus cannot be used to effectively detect miscommunications occurring in clinical practice.

### Data resources in patient safety research

Improving patient safety relies on evidence or PSE data in support of research studies and routine monitoring for rapid response. Table 1 lists typical data sources that are identifiable in the literature. We introduce each of them below and present the strengths and limitations of the sources in the table.

#### The American Society of Anesthesiologists (ASA) closed claims.

The ASA closed claims collect reports of malpractice and medical negligence, categorized as respiratory events, regional block, cardiovascular events, equipment, and medication [17]. The closed claims can study an extensive collection of relatively rare events (e.g., sentinel events on patient injuries); however, the data do not contain clinical contexts describing the whole process and may be biased to severe injuries.

#### Electronic health records.

EHR data contains diagnoses, procedures, vital signs, medication order history, medication administration record, computerized provider order entry, lab results, audit logs, and unstructured clinical notes [18]. Researchers leverage EHR data to identify medication administration errors (intravenous infiltrations, narcotic medication over-sedation, dosing errors, vasoactive medication, parenteral nutrition, and insulin), airway management adverse events, and adverse outcomes (e.g., ICU readmissions, central line-associated bloodstream infections). The EHR has the potential for enabling real-time identification of certain error types [18].

#### The Safety-related EHR Research (SAFER) Reporting.

Veterans Health Administration Informatics Patient Safety (IPS) Office established a platform for the voluntary reporting of EHR-related safety concerns [19]. IPS analyst reviews EHR logs and discusses the case with human factors specialists. The IPS team attempts to replicate the safety concern in the EHR by recreating the reported incident circumstances.

#### Patient safety events reported to Patient Safety Organizations.

Voluntary patient safety events (PSEs) reported by Patient Safety Organizations (PSO) provide nationwide and uniform protections in all US States and territories [20]. Each PSO and the providers it works with determine the scope or focus under the Patient Safety Act. The AHRQ Common Formats, a set of standardized definitions and formats for collecting, aggregating, and analyzing uniformly structured patient safety data for local, regional, and national learning. The Common Formats for event reporting include incidents, near misses, and unsafe conditions. The Common formats for Surveillance provide complementary information to improve event detection and the calculation of PSE rates.

#### FDA Adverse Event Reporting System (FAERS).

The FDA FAERS collected adverse drug events (ADEs) reported by healthcare professionals, consumers, and drug manufacturers through the MedWatch reporting program [21]. FAERS is a computer-based relational database made up of seven file packages arranged in tabular format. The file packages contain patient demographics, ADEs, drug therapy start and end dates, indications of use (diagnoses), names of the reported medications suspect for ADEs, and the type of outcome (life-threatening, hospitalization, or death) from the drug.

Patient safety data provides extensive evidence for care providers to identify root causes of PSE. Although the number of PSE prediction models relying on the safety data has been increasing, performances (accuracy) and capacities (focusing on a limited number of PSE) of those models in detecting real-time PSE are limited. Few models consider teamwork as a risk factor in predicting PSE. Telehealth as a novel mode providing healthcare services to patients creates opportunities to improve the performances and capacities of existing PSE prediction models and incorporating teamwork as an important risk factor.

### Telehealth to improving teamwork

Delivering care from off-site locations, tele-ICU is developed to address the increasing complexity of patients and the insufficient supply of intensivists in critical care hospitals in rural areas [6]. Since increasing the number of intensivists can improve care quality and patient safety, virtual intensivists offered via Tele-ICU can collaborate with bedside teams. Tele-ICU electronically integrates patients, bedside teams, tele-teams, and a variety of medical devices with all aspects of care. Tele-ICU platforms leverage algorithms to scrutinize patient data, combining physiological parameters with clinical risk factors to predict deterioration and provide decision support. The risk-prediction algorithms and push-notification dashboards embedded in the tele-ICU platforms enable one tele-ICU observer (intensivist) to watch multiple patients simultaneously where on-site support is unavailable. A tele-ICU observer has a higher probability of capturing the timing of adverse events, which are not realized by the bedside team. For example, when a depressed newborn begins to breathe spontaneously, an important physiologic time-point, a tele-ICU observer may better observe the time-point taking the advantage of a live and clear video stream without the involvement with resuscitation. Monitoring via live video stream provides an excellent opportunity to capture the time point of adverse events, which is particularly important for patient safety and clinical outcomes.

Tele-ICU observers have a clear vision of safety issues and can timely communicate the potential risks with the bedside teams. For instance, the miscommunication between clinicians in the above-mentioned example can be prevented by a tele-ICU observer’s involvement. The tele-ICU observer can remind the nurse or pharmacist that the patient should be administered as 50 ml hypertonic saline rather than 500 ml. Also, the observer can remind the nurse to check the sodium level in one hour. The involvement of tele-ICU enables both real-time medical error prevention and retrospective safety research. Tele-ICU provides an opportunity to videotape bedside team activities (miscommunication between the nephrology consultant, nurse, intensivist, and pharmacist) and medications (500 ml bag of hypertonic saline) administered to patients.

*Video tracking technologies* that have been implemented in telehealth platforms can be applied to the modern ICUs, which offers great potential for advances in unit operations, patient safety, staff efficiencies, and data analytics. Video recordings contain extensive information on team communication and patient health status (audiovisual). We can use audiovisual data to detect inefficient teamwork (e.g., miscommunications from medication prescribing to transcription, to dispensing, to administration), and measure the gap between executed and planned/trained teams.

Advanced *computer vision* (CV) technologies provide an excellent opportunity to analyze audiovisual data in an automated way [22]. CV technologies can capture bedside nurse interactions such as those occurring during bedside shift and hourly rounding [23]. Relying on CV, it becomes realistic to measure communications among clinicians and compare teamwork planned with those executed in clinical practice. For instance, the miscommunication between the nephrology physician, intensivist, and nurse in our example can be detected by using CV technologies. CV is used to recognize clinician and patient activity in clinics, which has been deployed in a range of ambient intelligence applications, continuous, non-invasive awareness of activity in a physical space where clinicians are offered assistance such as patient monitoring, automated documentation, and monitoring for protocol compliance.

**Figure 1-:**
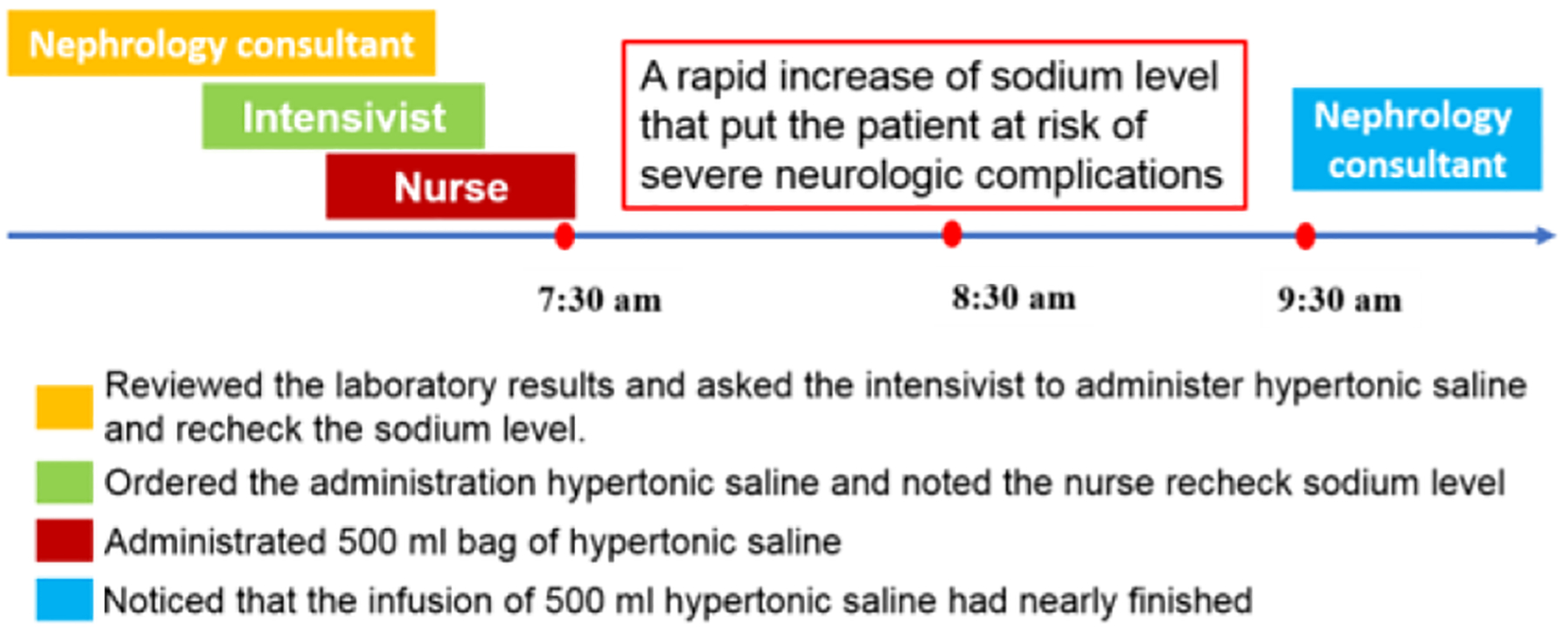
An ICU workflow for a hyponatremia patient.

The application of CV can also help with corresponding points across similar images (image registration), finding similar images (image retrieval), and reconstructing images [22]. In our example, as shown in Figure 1, CV might leverage visual data to detect the difference between 500 ml and 50 ml bags of hypertonic saline, resulting in an alert to the nurse automatically. CV models can detect the type of an object (e.g., hypertonic saline bag) in an image, and the segmentation of the object, which provides excellent space for the deployment of CV in a medical environment. Dozens of CV models have obtained US FDA and European CE approval, and commercial markets have begun to form. CV technology allows for more efficient audiovisual data processing, retrieving, and analyzing, which can reduce diagnostic and medication errors in ICUs. With the advanced CV technologies, bedside robots are promising to replace tele-ICU teams despite there is still a long way to go.

## Discussion

The availability of PSE data is paramount to tackle the issues in patient safety. The data collected from claims, Health IT, and voluntary safety event reports greatly contribute to the resource for patient safety research. However, the available data are insufficient to capture healthcare professional interactions, and clinician-patient activities occurring in physical space, which has posed barriers to patient safety research. Although observational studies can yield additional information on patient and clinician activities, it often focuses on a specific setting because it requires a substantial amount of manual effort. The advantages of tele-ICU provide an opportunity to capture audiovisual data and advance patient safety research into the era empowered by artificial intelligence (AI). Also, with the advanced CV technologies, AI-based safety protection tools are expected to be deployed in ICUs in near future.

Telehealth, video recordings, and CV provide excellent opportunities for the enhancement of patient safety (prospective and retrospective), however, there exist many barriers that need to be solved. First, there is a lack of strategies or protocols to link video recordings to existing PSE taxonomy (e.g., AHRQ common formats). Taxonomies need to be developed to categorize information retrieved from audio-visual data, especially for those related to teamwork. Second, although technologies in CV-based ambient intelligence are readily available for healthcare, there are perceived barriers and ethical issues in implementing telehealth in the ICU settings. The ICU settings are extremely complex, and clinicians are frequently mobile. Therefore, it is hard for telehealth platforms to capture the entire team activities surrounding specific patient care, which requires various handoffs. It might be feasible to start video tracking for an established workflow (e.g., transitioning patients from the operating room to post-anesthesia care unit, or team meetings) and monitor clinicians or team activities in that fixed scenario. Leveraging telehealth platforms to videotaping the entire ICU scenarios is a long journey. Moreover, the operational cost to install a telehealth platform ranges between $50,000 and $100,000 per ICU bed for the first year, covering hardware, installation, software licenses, staffing, and other operational expenses [6]. Therefore, it is less beneficial for healthcare organizations with small cash flow to implement such a platform. Third, there is a lack of mature video data standards to store, manage, and process massive video recordings yield from telehealth platforms from a technology perspective. High-Efficiency Video Coding (HEVC) standard enables the transmission and storage of a large amount of video much more practical [19]. Recently, Versatile Video Coding reduces data requirements by around 50% of the bit rate relative to HEVC, and video files can be transmitted twice as fast [24]. Also, concerns on privacy and confidentiality (HIPAA) on patients and staff should be adequately addressed. Regulations and policies need to be developed to protect patient and provider privacy.

## Conclusions

ICUs represent highly complex medical environments, using sophisticated technology and caring for a vulnerable patient population. They are breeding grounds for errors because interdependent components interact in unexpected ways. Miscommunication and a broken shared mental model are associated with medical error and mortality risk in ICUs. This perspective shows main patient safety and teamwork challenges in ICUs, and the effort made by national health organizations and patient safety research communities to enhance high-performing teamwork and patient safety. Finally, we discuss the excellent opportunities brought by telehealth and CV to solve the challenges to predict or prevent ICU safety events.

## Figures and Tables

**Table 1 –:** Data sources in support of patient safety research

Strengths	Limitations
**The American Society of Anesthesiologists (ASA) closed claims**	
The ability to study a large collection of relatively rare events; Extensive data including detailed clinical information on large collections of difficult intubations, pulmonary aspirations, central venous catheter complications, medication errors.	Bias towards more severe injuries; Failure to draw together all the evidence and consider its implications; Inadequate clinical notes impede the whole process;
**EHR systems derived data**	
Present a granular, precise, and comprehensive view of patients; Using standard terms represents diagnoses, procedures, labs, &medications; Supplement of incident reporting; Offer an automatic way to evaluate medication orders and administrations (readmissions, central line-associated infections).	EHRs do not include medication administrations during surgery and resuscitations. Medication orders do not have standard terms Free-text notes contain extensive patient safety information without standardization.
**The safety-related EHR research (SAFER) reporting database**	
Incident analysis and feedback to EHR developers can help mitigate future risks to patients. Investigate EHR-related adverse events, near-misses and report them to the national board using standardized methods. The distribution of EHR-related safety incidents is nationwide. Guided by an 8-dimension sociotechnical model	Only health IT-related reports. Minor or latent errors and near-misses may not be included. Self-reported data is biased to reporters’ recall and knowledge. Standardized data collection models not widely used. Limited access to Veterans Health Administration
**Patient safety events reported by patient safety organizations, AHRQ Common Formats**	
Harmonize across governmental health agencies and incorporate feedback from the private sector. Non-identifiable and aggregated data are stored in the Network of Patient Safety Databases (NPSD), Supporting Dashboards, Chartbooks, AHRQ’s National Healthcare Quality and Disparities Report.	A separate reporting process beyond clinical information systems. The Common Formats are evolving. Two versions in use. PSE in ICUs are scattered or may be under-reported as the formats do not specify ICU settings.
**FDA FAERS**	
A national repository of adverse drug events, A microcosm of drug treatment and outcomes. Drug names matched to RxNorm reference codes, Adverse drug events matched to MedDRA. Structured sections (excluding narratives) are publicly accessible.	Missing physiologic, psychologic, or demographic information. Signals detected from FAERS need to be validated by other sources such as EHRs and biomedical literature. Narratives for individual case reports can be requested.
